# The role of SARS-CoV-2-mediated NF-κB activation in COVID-19 patients

**DOI:** 10.1038/s41440-023-01460-2

**Published:** 2023-10-23

**Authors:** Qiaoqiao Zhou, Lei Zhang, Yanming Dong, Yuan Wang, Bin Zhang, Shiyi Zhou, Qing Huang, Tian Wu, Gongxuan Chen

**Affiliations:** 1 School of Chemistry and Life Sciences, Hubei University of Education, Wuhan, Hubei 430205 PR China; 2 Hubei Key Laboratory of Purification and Application of Plant Anticancer Active Ingredients, School of Chemistry and Life Sciences, Hubei University of Education, Wuhan, Hubei 430205 PR China; 3 Hubei Environmental Purification Material Science and Engineering Technology Research Center, Hubei University of Education, Wuhan, 430205 China; 4 School of Life Sciences, Hubei University, Wuhan, 430062 China; 5 School of Basic Medicine, Hubei University of Arts and Science, Xiangyang, 441053 China

**Keywords:** COVID-19, SARS-CoV-2, NF-κB activation, Inflammatory response, COVID-19 drugs

## Abstract

The SARS-CoV-2 pandemic, now in its third year, has had a profound impact on public health and economics all over the world. Different populations showed varied susceptibility to this virus and mortality after infection. Clinical and laboratory data revealed that the uncontrolled inflammatory response plays an important role in their poor outcome. Herein, we summarized the role of NF-κB activation during SARS-CoV-2 invasion and replication, particularly the angiotensin-converting enzyme 2 (ACE2)-mediated NF-κB activation. Then we summarized the COVID-19 drugs’ impact on NF-κB activation and their problems. A favorable prognosis is linked with timely treatment with NF-κB activation inhibitors, such as TNFα, IL-1β, and IL-6 monoclonal antibodies. However, further clinical researches are still required to clarify the time window, dosage of administration, contraindication, and potential side effects of these drugs, particularly for COVID-19 patients with hypertension, hyperglycemia, diabetes, or other chronic diseases.

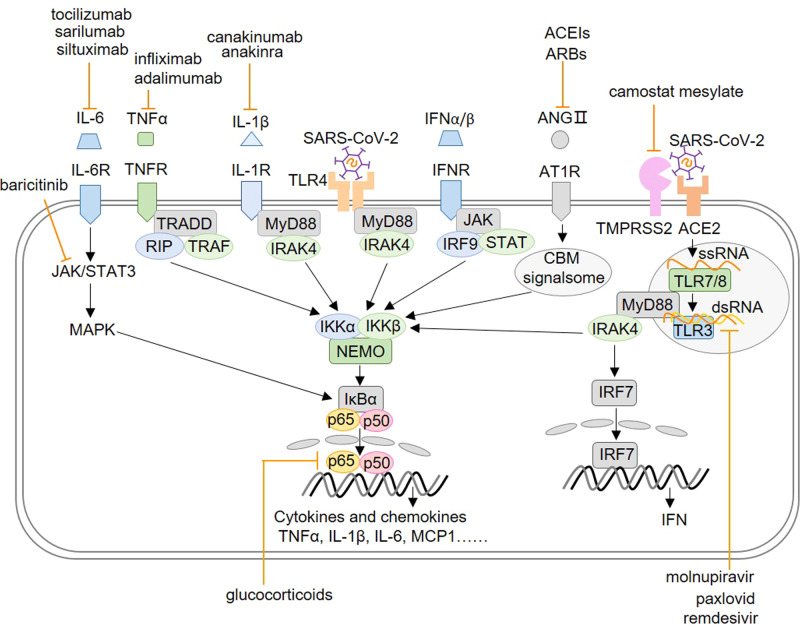

## Introduction

The global coronavirus disease 2019 (COVID-19) pandemic has threatened public health for more than three years. Clinical symptoms of COVID-19 range from mild respiratory illness to severe pneumonia, multiple organ failure, and even death [1]. Most COVID-19 patients show moderate symptoms or even no symptoms. However, a significant portion of individuals could develop acute pneumonia and require hospitalization [2]. The leading death causes of COVID-19 include acute respiratory distress syndrome (ARDS), multiple organ failure, respiratory failure, shock, and so on [3].

Due to the liberalization of epidemic prevention and control policies, the main policy to deal with the epidemic has changed from “prevention” to “treatment”. Therefore, it is particularly important to select appropriate drugs for patients with different severity and disease stages. In the early stages of a virus invasion, a strong enough inflammatory response is essential to resist the virus [4]. While an uncontrolled inflammatory response or even an inflammatory factor storm may be damaging or even fatal [5, 6].

Although a lot of clinical studies have demonstrated that individuals with hypertension are more susceptible to COVID-19 and have a worse prognosis, however little emphasis has been paid to the role that NF-κB activation plays in its causes, development, diagnosis, and therapeutics. Therefore, this review summarized the changes in NF-κB activation in the renin–angiotensin–aldosterone system (RAAS) during severe acute respiratory syndrome coronavirus 2 (SARS-CoV-2) infection and chronic treatment of angiotensin-converting enzyme inhibitors (ACEIs) and angiotensin receptor blockers (ARBs). In particular, we concentrated on the COVID-19 drugs’ effects on NF-κB activation and their problems, in effort to provide some theoretical basis for regulating uncontrolled inflammatory response.

## NF-κB activation

**Fig. 1:**
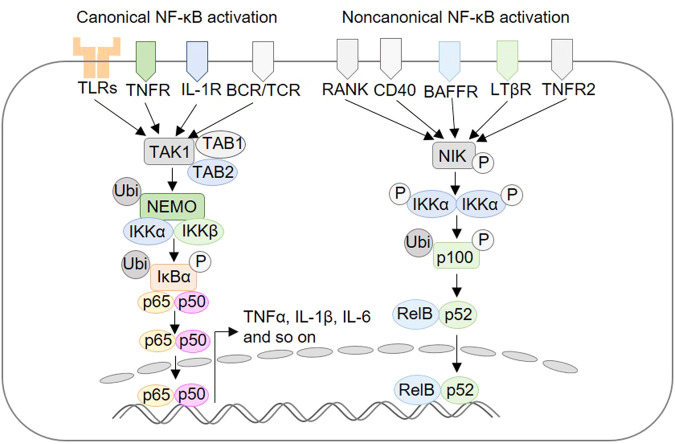
The canonical and non-canonical NF-κB activation signaling pathway. In the canonical NF-κB activation pathway, IKKβ and IKKγ are required and the NF-κB dimers are usually p65/p50 or p50/c-Rel dimers. In the non-canonical NF-κB activation pathway, however, phosphorylation of NIK and activation of IKKα are required, and the NF-κB dimers are usually RelB/p52 dimer

The inflammatory response depends on the activation of multiple transcription factors, of which the nuclear factor-κB (NF-κB) plays an important role. Mammalian NF-κB includes five members: RelA (p65), RelB, c-Rel, p50, and p52. NF-κB is a heterodimer. The NF-κB complex is present in the cytoplasm and is inactive in most cells due to its binding to suppressor IκB protein [7]. When the cell is stimulated, the inhibitor of NF-κB (IKK) phosphorylates and degrades IκB. Then the NF-κB complex is released and enters the nucleus to regulate target gene expression. As shown in Fig. 1, degradation of IκB is a marker of activation of NF-κB in most cases, henceforth activation of NF-κB can be divided into canonical and non-canonical pathways according to the different signaling pathways leading to the degradation of IκB [8]. In the canonical pathway, IKKβ and IKKγ are required to activate the NF-κB dimer, while IKKα is not required. For example, both p50/p65 and p50/c-Rel dimer are activated by the canonical pathway. Unlike the canonical pathway, the non-canonical NF-κB activation needs phosphorylation of NF-κB inducing kinase (NIK) and activation of IKKα, but not degradation of IκB. Then p100 is partially hydrolyzed to form mature p52 via ubiquitin-proteasome pathway. Stimuli and receptors activating the canonical and non-canonical pathways are also different. The activation of the canonical pathway is usually through tumor necrosis factor receptor (TNFR1), interleukin 1 receptor (IL-1R) and toll-like receptors (TLRs), while the non-canonical pathway is mostly activated through the receptor activator of NF-κB (RANK) of B lymphocytes, CD40, B-cell activating factor receptor (BAFFR), lymphotoxin β receptor (LTβR) and TNFR2 [9]. Moreover, the receptors that activate non-canonical pathways could also activate canonical pathways, so the two activation pathways can partially overlap. Meanwhile, the gene expression profiles targeted by the two are different. Canonical NF-κB activation is the main pathway of immune and inflammatory response, while non-canonical NF-κB activation is associated with lymphatic organogenesis.

With corresponding receptors for each type of stimulus, as seen in Fig. 1, NF-κB might react to a wide range of stimuli. Following ligand binding, the receptors start a series of events by recruiting their associated downstream adaptor proteins. After entering the nucleus, the NF-κB binds with the DNA and produces a variety of effectors. Abnormal NF-κB activation has been associated with a variety of diseases, including inflammatory diseases, immune deficiency, diabetes, atherosclerosis and tumors. Therefore, the NF-κB activation must be strictly controlled.

## The ACE2-mediated NF-κB activation during SARS-CoV-2 invasion

COVID-19 is caused by severe acute respiratory syndrome coronavirus 2 (SARS-CoV-2). The genome of this virus is a single stranded positive-sense RNA (+ssRNA) of approximately 29.9 kb [10]. And this genome encodes four structural proteins, sixteen non-structural proteins and nine accessory proteins [10]. The sixteen non-structural proteins (nsp1 to nsp16) are mainly responsible for the process of viral replication, whose coding genes are located in the 5’ end and make up two-thirds of the entire genome [10]. The four structural proteins include spike protein (S), membrane protein (M), envelope protein (E), and nucleocapsid protein (N) [10]. For the assembly of viral particles, the M protein and the E protein are necessary. The N protein forms the outside of the genome. Additionally, the S protein could bind with the receptor on the host cell. While the accessory proteins, open reading frame 3a (ORF3a), ORF3b, ORF6, ORF7a, ORF7b, ORF8, ORF9b, ORF9c, and ORF10, whose coding genes are inserted in the four structural proteins genes, are thought to play significant roles in viral pathogenesis [10].

**Fig. 2:**
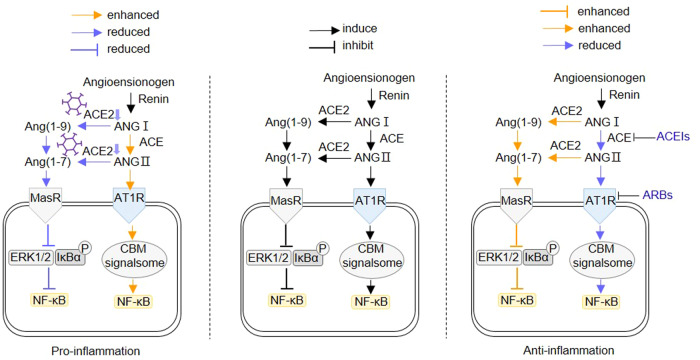
The mechanism of ACE2-mediated NF-κB activation by anti-hypertension drugs and SARS-CoV-2 invasion. (middle)The inactive angiotensinogen is catalyzed to form the ANG I by the renin. Then the ANG I is converted to the ANG II by ACE, which binds to its receptor AT1R to and lead to increased blood pressure, enhanced NF-κB activation and so on. Moreover the ACE2 could catalyze the ANG I to Ang (1–9), and ANG II to Ang(1–7). Ang (1–9) could also be converted to Ang(1–7), which binds to its receptor MAS to lower blood pressure, inhibit NF-κB activation and so on. (right) Chronic treatment of ACEIs and ARBs inhibits ACE2 expression and conversion from ANG II to Ang (1–7), resulting in inhibited ANG II/AT1R and enhanced Ang(1–7)-MasR axis. (left) SARS-CoV-2 binding significantly reduces ACE2 catalytic activity through competitive inhibition, leading to increased ANG II/AT1R and inhibited Ang(1–7)-MasR axis

When SARS-CoV-2 enters the cells by its S protein which is composed of two subunits S1 and S2. The S1 binds with the extracellular domain of angiotensin-converting enzyme 2 (ACE2), and the transmembrane protease serine 2 (TMPRSS2) cuts the S protein to produce S1 and S2 subunits, facilitating the S2-induced membrane infusion and viral invasion [10]. Compared with the SARS-CoV, the high spreading efficiency of SARS-CoV-2 results from its high affinity of S1 with ACE2. As shown in Fig. 2, ACE2 is a crucial enzyme of the RAAS which regulates blood pressure and electrolyte balance. Firstly, angiotensinogen is produced by the liver and secreted into the plasma. Then the inactive angiotensinogen is catalyzed to form angiotensin I (ANG I) by the renin which is secreted by the kidneys. The ANG I is also inactive unless it is catalyzed to form the angiotensin II (ANG II) by angiotensin-converting enzyme (ACE) [11]. ANG II binds to its receptor angiotensin type 1 receptor (AT1R), which could recruit adaptors to form CARMA3·BCL 10·MALT1 complex (CBM signalosome) [12]. The CBM signalosome is composed of CARD-containing MAGUK protein 3 (CARMA3), which is also named caspase recruitment domain protein 10(CARD10), B-cell lymphoma 10 (BCL 10) and mucosa-associated lymphoid tissue (MALT1) [13]. Then the CBM signalosome activates the IκB kinase complex, causing IκB to be phosphorylated at Ser 32/36 and degraded by ubiquitin-proteasome pathway [13, 14]. Finally released NF-κB subunits translocate into the nuclease and induce the expression of inflammatory cytokines and chemokines in the pulmonary epithelial cells.

As shown in Fig. 2, the ACE2 catalyzes the ANG I to Ang (1–9), and ANG II to Ang(1–7) through cutting the C-terminal amino acid residues. Ang (1–9) could also be converted to Ang(1–7), which binds to the MAS receptor to inhibit the NF-κB activation by inhibiting the ERK1/2 activation and the phosphorylation of IκBα [15, 16]. The SARS-CoV-2 binding could reduce ACE2 catalytic activity through competitive inhibition, leading to enhanced ANG II-AT1R axis and reduced ACE2- MAS axis, both of which would enhance NF-κB activation.

For hypertensive COVID-19 patients, the unstable inflammation balance may contribute to a higher susceptibility to inflammatory dysfunction or even inflammatory cytokines storm. Clinical data showed that the chronic treatment of angiotensin-converting enzyme inhibitors (ACEIs) and angiotensin receptor blockers (ARBs) facilitated their prognosis. As shown in Fig. 2, both drugs could inhibit the ANG II-AT1R axis and enhance the ACE2-MAS axis, inhibiting NF-κB activation [10]. The suppressed NF-κB activation effectively decreases the production of pro-inflammatory cytokines in lung alveolar cells and protects the lungs. Yang et al. found that ARBs/ACEIs treatment also resulted in a marginally lower death rate and fewer critical cases in a retrospective study of 126 COVID-19 patients with hypertension [17]. Similarly, Batiha et al. also found that hypertensive COVID-19 patients on ACEIs/ARBs treatments had lower mortality compared to the other anti-hypertensive medications in a retrospective analysis of 1128 cases [18]. Especially when they were used in combination with tocilizumab, the therapeutic effect is better than tocilizumab treatment alone, which could also reduce its effective dosage [19]. In addition to hypertensive COVID-19 patients, it was proposed that ACEIs/ARBs also reduce the risk of disease severity among elderly and diabetes COVID-19 patients [20].

## SARS-CoV-2 associated NF-κB activation

**Fig. 3:**
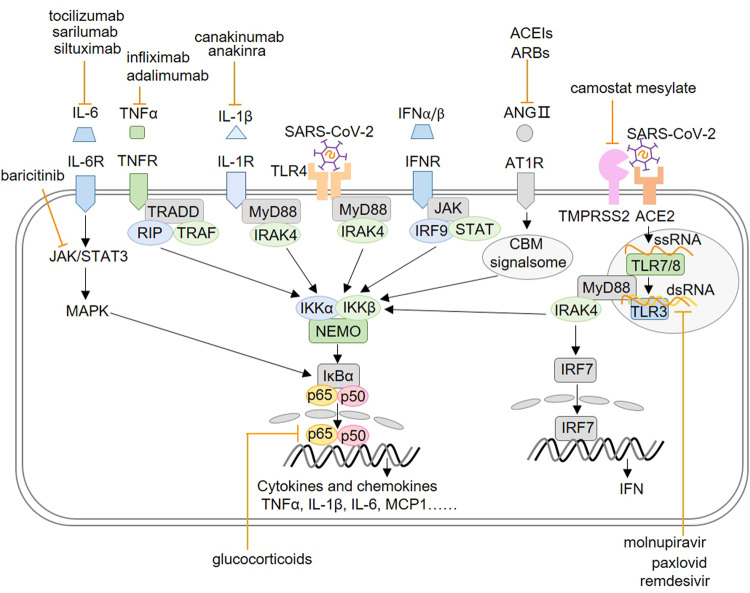
SARS-CoV-2 induces inflammatory dysfunction through over-stimulating NF-κB activation. During invading, SARS-CoV-2 occupies ACE2 and inhibits its activity, which leads to increased ANG II/AT1R and inhibited Ang(1–7)-MasR axis, resulting in enhanced NF-κB activation. During invasion SARS-CoV-2 could be recognized by TLR4. After invasion, uncoated virus single strand RNA could be recognized by TLR7/8. During RNA replication the double strand RNA could be recognized by TLR3. Some anti-virus drugs inhibit the invasion SARS-CoV-2 replication, such as remdesivir, molnupiravir and paxlovid. These TLRs could induce the NF-κB activation which induces the expression of down-stream pro-inflammatory cytokines and chemokines, such as TNFα, IL-1β, IL-6, MCP1 and so on. All of these molecules could further enhance the NF-κB activation through the NF-κB self-activation or other pathways. IL-6/IL-6R monoclonal antibodies or JAK inhibitors suppress NF-κB activation by inhibiting IL-6/JAK/STAT3 signaling, such as tocilizumab, sarilumab, siltuximab, clazakizumab, baricitinib, ruxolitinib and tofacitinib

It was found that the severe COVID-19 cases showed stronger NF-κB activation, as well as higher levels of C-reactive protein (CRP), interleukin-6 (IL-6), ferritin and D-dimer [21]. The SARS-CoV-2 could also stimulate the NF-κB activation through several ways in addition to enhanced ANG II/AT1R and inhibited Ang(1–7)-MasR axis. As shown in Fig. 3, SARS-CoV-2 could be recognized by TLRs to induce the NF-κB activation. During the invasion the virus is recognized by TLR4 on the plasma membrane. After the invasion, uncoated virus single strand RNA (ssRNA) could be recognized by TLR7/8 on the endosome membrane [22]. During RNA replication the double-strand RNA (dsRNA) could be recognized by TLR3 in the endosome [22]. These TLRs could recruit the toll-like receptor adapter protein myeloid differentiation primary response 88 (MyD88), which recruits the interleukin-1 receptor-associated kinase 4 (IRAK4) [22]. Then IRAK4 activates the IKK complex and degrade IκBα, releasing the inhibited NF-κB. The activated NF-κB enters the nucleus and enhances the expression of down-stream pro-inflammatory cytokines and chemokines, such as TNFα, IL-1β, IL-6, MCP1 and so on [21]. Furthermore, other studies revealed that a few viral accessory proteins could also induce NF-κB activation. Su et al. discovered that ORF7a activated NF-κB, inducing expression of pro-inflammatory cytokines such as IL-1α, IL-1β, IL-6, IL-8, IL-10, TNFα, and IFNβ [23]. ORF3a could also induce pro-inflammatory cytokines by enhancing the interaction between IKKβ and NEMO, resulting in increased NF-κB activation [24]. In addition to ORF proteins, nsp1, nsp3a, and nsp7a can also over-activate the NF-κB, which induces cytokines storm and contributes to the multi-organ damage [22].

Furthermore, oxidative stress (OS), which is caused by an excessive amount of reactive oxygen species (ROS) production, also contributes to the severity of COVID-19. Firstly, by inhibiting tyrosine phosphatases, H_2_O_2_ might directly activate a number of protein kinase signaling pathways, including ERK1/2 and Akt, which then cause NF-κB activation. Secondly, OS can cause lung macrophages to produce oxidized phospholipid, which binds to TLR4, recruits tumor necrosis factor receptor-associated factor 6 (TRAF6) and TRIF, and causes excessive NF-κB activation [25]. In addition to TLR4, TLR7 could recognize ssRNA, induce p47phox phosphorylation and activate nicotinamide adenine dinucleotide phosphate (NADPH) oxidase 2 (NOX2), triggering OS during SARS-CoV-2 infection, which led to NF-κB activation [26]. In addition to TLR-NF-κB signaling pathways linked to SARS-CoV-2, ANG II-induced-OS also contributes to acute lung injury. Invading SARS-CoV-2 inhibits ACE2 activity and boosts the binding of ANG II to AT1R, which leads to OS via inducing NOXs [27]. Dysregulated ROS then activate the NF-κB to cause the release of pro-inflammatory cytokines by stimulating the ERK1/2 and p38 pathways [27]. As a result, patients with COVID-19 who have higher basic ROS—such as the elderly and those who have hypertension—are more prone to experience severe symptoms [27].

In addition to NF-κB translocation, the SARS-CoV-2 also induces IRF7 translocation and production of interferon [28]. In the early stage, SARS-CoV-2-induced IFN response is essential to resist the invading virus, and prompt supplementation of IFN would prevent severe pathogenic reaction before the peak of virus replication [29]. While a delayed type I IFN (IFN-I) response leads to rapid virus replication [30]. After that, inflammatory monocyte-macrophages (IMMs) are recruited to the lungs, and aberrant NF-κB activation occurs in the pulmonary epithelial cells and immune cells. As shown in Fig. 3, produced cytokines enhance the NF-κB activation through the NF-κB self-activation or other pathways. TNFα binds with TNFR and recruits TNFRSF1A associated via death domain (TRADD), tumor necrosis factor receptor -associated factor (TRAF), and receptor-interacting protein (RIP), which activates IKK complex to induce NF-κB activation. IL-1β binds with IL-1R, recruits MyD88, IRAK4 and phosphate the IKK complex to activate NF-κB. IL-6 induces NF-κB activation via IL-6/JAK/STAT3 and mitogen-activated protein kinases (MAPKs) signaling pathways. Heightened inflammatory cytokines and chemokines increase endometrial permeability and recruit more immune cells, leading to an enlarged inflammatory response or even an inflammatory cytokine storm [31]. Karki et al. proposed that TNFα and IFN-II synergistically promoted cell death during SARS-CoV-2-induced sepsis and multiple organ failure in mice [32]. It has also been reported that the constitutive activation of NF-κB and enhanced TNFα, IL-1β, and IL-6 levels is a ubiquitous phenomenon among various cell types in severe COVID-19 patients [31]. At this stage, controlling constitutive NF-κB activation is an important anti-inflammatory therapeutic strategy, especially for the elderly, hypertensive or hyperglycemic COVID-19 patients [33].

## The effect on NF-κB activation of COVID-19 drugs

According to Ma et al. suppressing the NF-κB activity could effectively inhibit virus replication and the release of pro-inflammatory cytokines [34]. As shown in Fig. 3 and Table 1, a range of COVID-19 drugs are evaluated, which could affect the NF-κB activation [35]. The nucleotide analog drugs suppress the NF-κB activation by inhibiting the viral RNA-dependent RNA polymerase (RdRp), such as remdesivir, molnupiravir and paxlovid [36, 37]. The latter two drugs have already been approved for the treatment of COVID-19. The glucocorticoids (methylprednisolone and dexamethasone) suppress the NF-κB activation by disrupting the NF-κB’s binding with its DNA responsive sequences [38, 39]. In addition to traditional anti-inflammatory drugs glucocorticoids, several rheumatoid arthritis drugs are also used to control the expansion of inflammatory response. The IL-6/IL-6R monoclonal antibody suppresses the NF-κB activation by disrupting the interaction between IL-6 and its receptors, such as tocilizumab, sarilumab, siltuximab and clazakizumab [40]. The JAK inhibitors also suppress the NF-κB activation by disrupting the complete JAK/STAT/NF-κB signaling pathways, such as baricitinib, ruxolitinib, and tofacitinib [41]. It was proposed that baricitinib reduced 28-day and 60-day mortality when used in addition to the current standard treatment [41]. TNFα inhibitors antagonize TNFα-induced NF-κB activation, such as TNFα monoclonal antibodies infliximab and adalimumab. Both were associated with a lower probability of hospitalization and severe COVID-19 [42]. For anakinra and canakinumab, the IL-1α/β inhibitors, suppress the NF-κB activation by disrupting the interaction between IL-1α/β with IL-1R. It was found that the early start of treatment with anakinra in patients hospitalized with moderate and severe COVID-19 significantly reduced the risk of worse clinical outcomes [43]. Clinical data showed that these anti-inflammatory drugs lower the mortality of the severe COVID-19 patients with ARDS [44]. Additionally, the TMPRSS2 inhibitor camostat mesylate reduces NF-κB activation by preventing SARS-CoV-2 fusion with the host plasma membrane and viral replication [45, 46].

**Table 1:** COVID-19 related drugs and their targets in NF-κB activation

Drug		Effect on NF-κB activation	References
1	Remdesivir/molnupiravir	Suppress viral replication by specifically inhibiting RdRp as a nucleotide analogue	[36]
2	Paxlovid	Suppress viral replication by specifically inhibiting the chemotripsine-like protease of SARS-CoV-2	[37]
3	Methylprednisolone/ dexamethasone	Suppress NF-κB activation by disrupting the NF-κB’s binding with its DNA sequences	[38, 39]
4	Tocilizumab/sarilumab/ siltuximab/clazakizumab	Suppress IL-6R-induced NF-κB activation by disrupting the interaction between IL-6 and IL-6R	[40]
5	Infliximab/adalimumab	Suppress TNFR-induced NF-κB activation by disrupting the interaction between TNFα and TNFR	[42]
6	Anakinra/canakinumab	Suppress IL-1R-induced NF-κB activation by disrupting the interaction between IL-1α/β with IL-1R	[43]
7	Baricitinib/ruxolitinib/tofacitinib	Disrupt the JAK/STAT/NF-κB signaling pathways by inhibiting JAK1/2 activation	[41]
8	Camostat mesylate	Suppress NF-κB activation by inhibiting SARS-CoV-2 fusion by inhibiting TMPRSS2	[45, 46]
9	Metformin	Suppresses NF-κB activation by inhibiting p38 activation and ANG II-AT1R axis	[47]
10	ACEIs/ARBs	Suppress NF-κB activation via ANG II-AT1R and ACE2-MAS signaling	[22]
11	Fluvoxamine	Suppress NF-κB activation by inhibiting PI3K/AKT signaling through GSK1	[48]
12	Chloroquine/hydroxychloroquine	Suppress NF-κB activation by inhibiting PI3K/AKT signaling	[49]
13	Aspirin	Suppress NF-κB activation by inhibiting both type 1 and type 2 cyclooxygenase and reducing ROS generation	[50]
14	N-acetylcysteine	Suppress NF-κB activation by lowering ROS by replenishing intracellular reduced glutathione	[51, 52]
15	Glutathione	Suppress NF-κB activation by lowering ROS generation	[53]
16	Vitamin C	Suppress NF-κB activation by lowering ROS generation	[53]

In addition to inhibition of the TNFα, IL-1β and IL-6 induced NF-κB activation, several COVID-19 drugs inhibit the NF-κB activation through disrupting the crosstalk signaling, such as MAPK(JNK/ERK/p38), ANG II-AT1R and ACE2-MAS signaling pathways [22]. For example, metformin suppresses NF-κB activation via inhibiting p38 activation and ANG II-AT1R axis [47]. Above mentioned ACEIs and ARBs suppress the NF-κB activation via ANG II-AT1R and ACE2-MAS signaling. The antipsychotic drug fluvoxamine suppresses the NF-κB activation for mild COVID-19 patients by inhibiting PI3K/AKT signaling through glycogen synthase kinase 1 (GSK1) [48]. Chloroquine/hydroxychloroquine acts as a TNFR antagonist and PI3K/AKT signaling inhibitor to suppress the NF-κB activation, while the severe COVID-19 patients didn’t benefit from chloroquine/ hydroxychloroquine [49]. Moreover, several drugs lower the NF-κB activation by regulating the cellular redox balance. For example, aspirin inhibits NF-κB activation by inhibiting both type 1 and type 2 cyclooxygenase to lower virus-induced ROS [50]. Researches showed that aspirin increased the rate of being discharged alive within 28 days even though it didn’t reduce mortality or the risk of progressing to invasive mechanical ventilation or death [50]. Although N-acetylcysteine lowers virus-induced ROS by replenishing intracellular reduced glutathione through providing cysteine, severe COVID-19 patients didn’t benefit from it [51]. However, N-acetylcysteine was recommended to treat SARS-CoV-2-induced inflammatory response combined with pirfenidone for maximum efficiency [52]. In addition to N-acetylcysteine, oxidant therapies also use glutathione (GSH) and vitamin C [53]. For instance, GSH inhalation reduced excessive ROS and protected the respiratory tract [53]. Clinical studies also demonstrated that infusion of vitamin C prevented OS and reduced acute lung injury and ARDS by inhibiting NF-κB driven cytokine storm [53].

## Problems with drugs targeting NF-κB activation in COVID-19

To stop irreversible ARDS and multi-organ dysfunction that follows COVID-19 from developing and progressing, the hyper-inflammatory response must be promptly controlled. For example, dexamethasone was used to inhibit the cytokine storms induced by COVID-19. In a retrospective study of 6425 cases, it was found that dexamethasone for 10 days resulted in lower 28-day mortality than non-dexamethasone group in hospitalized patients receiving respiratory support [54]. However, they also pointed out that dexamethasone did not provide any benefit among patients who were not receiving respiratory support [54]. On the other hand, the survivors may show long-term sequelae and a delayed viral clearance after high-dose corticosteroid treatment [55]. Even though IL-6 inhibitors could be useful in severe or critical COVID-19 patients, appropriate timing and dosage, monotherapy or combination therapy, and proper side effect management must be noticed regarding the clinical administration of these drugs, as IL-6 could either inhibit or increase viral replication and function [56]. Even though most studies have shown the beneficial effects of metformin in COVID-19 patients, some studies have reported an increased risk of acidosis and aggravation in COVID-19 patients using metformin [57]. As metformin is not an appropriate choice in patients with severe respiratory distress, renal impairment, or heart failure and contraindications to metformin must be addressed before administration [58, 59]. In the early COVID-19 pandemic, the chloroquine/hydroxychloroquine was also used to treat SARS-CoV-2 infection [49]. However, a series of studies showed that both of drugs not only failed to benefit the patient, but also increased the frequency of ventricular arrhythmias [49]. In summary, the safety and effectiveness of drugs affecting NF-κB activation still need to be carefully approved for COVID-19 treatment. And a series of research highlights the importance of paying attention to pre-existing comorbidities in drug selection. More clinical researches are still needed to clarify the time window, dosage of administration, the safety profile and potential side effects of these drugs [60].

## Conclusion

COVID-19 patients with hyperglycemia and hypertension showed worse prognosis and higher mortality [44]. Therefore, blood glucose and blood pressure must be properly monitored and managed to improve prognosis and survival rates. According to the World Health Organization (WHO), about one-quarter of the population have hypertension worldwide in 2022 [61]. In developing countries, the proportion is even higher [61]. About 422 million people worldwide have diabetes, and the majority live in low-and middle-income countries [62]. Moreover, many more people are suffering hyperglycemia and developing type 2 diabetes [62]. Henceforth rational drug treatment and selecting appropriate prognostic indicators may improve the outcome and survival of COVID-19 patients with hyperglycemia and hypertension. In addition to the medications that treat the symptoms, both hypoglycemic drugs and hypotensive drugs were recommended for the clinical treatment of COVID-19 infection for them.

In this review, we summarized the potential underlying mechanisms of the increased susceptibility and poorer prognosis for COVID-19 patients with hypertension as well as the COVID-19 drugs in relation to NF-κB activation. For COVID-19 patients with hyperglycemia and hypertension, however, more clinical trials and prospective studies are still required to demonstrate the efficacy of these drugs and the combination regimen. On the other hand, as more and more people are recovering from COVID-19, growing reports have raised the issue of “long COVID-19”, which is defined as emergent novel complications after viral infection for several weeks or months, including fatigue, hair loss, headache, attention disorder, deteriorating brain, and so on. Some long COVID-19 drugs also affect NF-κB activation [63]. For instance, vitamin D3 increases IκBα, which suppresses NF-κB activation [64]. While the acute COVID-19 symptoms were the main focus of this review, the inflammatory response and drugs of long COVID-19 were not adequately discussed. Until now, although a lot of studies have shown that NF-κB-mediated inflammatory dysfunction induced by SARS-CoV-2 contributed to worse prognosis and higher mortality of COVID-19 patients with hypertension, the underlying mechanism was still unclear. More researches are still needed on the effect of the anti-COVID-19 drugs.

## References

[1] Zhang J, Wang Z, Wang X, Hu Z, Yang C, Lei P (2021). Risk factors for mortality of COVID-19 patient based on clinical course: a single center retrospective case-control study. Front Immunol.

[2] Chang WT, Toh HS, Liao CT, Yu WL (2021). Cardiac involvement of COVID-19: a comprehensive review. Am J Med Sci.

[3] Jayk Bernal A, Gomes da Silva MM, Musungaie DB, Kovalchuk E, Gonzalez A, Delos Reyes V (2022). Molnupiravir for oral treatment of Covid-19 in nonhospitalized patients. N. Engl J Med.

[4] Pasrija R, Naime M (2021). The deregulated immune reaction and cytokines release storm (CRS) in COVID-19 disease. Int Immunopharmacol.

[5] Huang Q, Wu X, Zheng X, Luo S, Xu S, Weng J. Targeting inflammation and cytokine storm in COVID-19. Pharmacol Res. 2020;234:12865–75. 10.1016/j.phrs.2020.105051 PMC7320704 32603772

[6] Jose RJ, Manuel A (2020). COVID-19 cytokine storm: the interplay between inflammation and coagulation. Lancet Respir Med.

[7] Khongthong P, Roseweir AK, Edwards J (2019). The NF-KB pathway and endocrine therapy resistance in breast cancer. Endocr Relat Cancer.

[8] Zinatizadeh MR, Schock B, Chalbatani GM, Zarandi PK, Jalali SA, Miri SR (2021). The Nuclear Factor Kappa B (NF-kB) signaling in cancer development and immune diseases. Genes Dis.

[9] Roberti A, Chaffey LE, Greaves DR (2022). NF-κB Signaling and Inflammation-Drug Repurposing to Treat Inflammatory Disorders?. Biol(Basel).

[10] Beyerstedt S, Casaro EB, Rangel ÉB (2021). COVID-19: angiotensin-converting enzyme 2 (ACE2) expression and tissue susceptibility to SARS-CoV-2 infection. Eur J Clin Microbiol Infect Dis.

[11] Ruocco G, Feola M, Palazzuoli A (2020). Hypertension prevalence in human coronavirus disease: the role of ACE system in infection spread and severity. Int J Infect Dis: IJID: Off Publ Int Soc Infect Dis.

[12] McAllister-Lucas LM, Jin X, Gu S, Siu K, McDonnell S, Ruland J (2010). The CARMA3-Bcl10-MALT1 signalosome promotes angiotensin II-dependent vascular inflammation and atherogenesis. J Biol Chem.

[13] Okamoto H, Ichikawa N (2021). The pivotal role of the angiotensin-II-NF-κB axis in the development of COVID-19 pathophysiology. Hypertens Res.

[14] Pérez de Diego R, Sánchez-Ramón S, López-Collazo E, Martínez-Barricarte R, Cubillos-Zapata C, Ferreira Cerdán A (2015). Genetic errors of the human caspase recruitment domain-B-cell lymphoma 10-mucosa-associated lymphoid tissue lymphoma-translocation gene 1 (CBM) complex: Molecular, immunologic, and clinical heterogeneity. J Allergy Clin Immunol.

[15] Huang W, Cao Y (2019). Activating Mas receptor protects human pulmonary microvascular endothelial cells against LPS-induced apoptosis via the NF-kB p65/P53 feedback pathways. J Cell Physiol.

[16] Zhang X, Jia F, Ma W, Li X, Zhou X (2022). DAD3 targets ACE2 to inhibit the MAPK and NF-κB signalling pathways and protect against LPS-induced inflammation in bovine mammary epithelial cells. Vet Res.

[17] Yang G, Tan Z, Zhou L, Yang M, Peng L, Liu J (2020). Effects of Angiotensin II Receptor Blockers and ACE (Angiotensin-Converting Enzyme) Inhibitors on Virus Infection, Inflammatory Status, and Clinical Outcomes in Patients With COVID-19 and Hypertension: A Single-Center Retrospective Study. Hypertension.

[18] Batiha GE, Gari A, Elshony N, Shaheen HM, Abubakar MB, Adeyemi SB (2021). Hypertension and its management in COVID-19 patients: The assorted view. Int J Cardiol Cardiovasc Risk Prev.

[19] Guan WJ, Zhong NS (2020). Clinical Characteristics of Covid-19 in China. Reply N. Engl J Med.

[20] Gupta R, Hussain A, Misra A (2020). Diabetes and COVID-19: evidence, current status and unanswered research questions. Eur J Clin Nutr.

[21] Kircheis R, Haasbach E, Lueftenegger D, Heyken WT, Ocker M, Planz O (2020). NF-κB pathway as a potential target for treatment of critical stage COVID-19 patients. Front Immunol.

[22] Attiq A, Yao LJ, Afzal S, Khan MA (2021). The triumvirate of NF-κB, inflammation and cytokine storm in COVID-19. Int Immunopharmacol.

[23] Su CM, Wang L (2021). Activation of NF-κB and induction of proinflammatory cytokine expressions mediated by ORF7a protein of SARS-CoV-2. Sci Rep.

[24] Nie Y, Mou L, Long Q, Deng D, Hu R, Cheng J (2023). SARS-CoV-2 ORF3a positively regulates NF-κB activity by enhancing IKKβ-NEMO interaction. Virus Res.

[25] Delgado-Roche L, Mesta F (2020). Oxidative stress as key player in severe acute respiratory syndrome coronavirus (SARS-CoV) infection. Arch Med Res.

[26] Rochette L, Ghibu S (2021). Mechanics insights of alpha-lipoic acid against cardiovascular diseases during COVID-19 infection. Int J Mol Sci.

[27] de Oliveira AA, Priviero F, Lima VV, Webb RC, Nunes KP (2021). COVID-19 and ROS storm: what is the forecast for hypertension. Am J Hypertens.

[28] Hirano T, Murakami M (2020). COVID-19: a new virus, but a familiar receptor and cytokine release syndrome. Immunity.

[29] Lowery SA, Sariol A, Perlman S (2021). Innate immune and inflammatory responses to SARS-CoV-2: Implications for COVID-19. Cell Host Microbe.

[30] Khadke S, Ahmed N, Ahmed N, Ratts R, Raju S, Gallogly M (2020). Harnessing the immune system to overcome cytokine storm and reduce viral load in COVID-19: a review of the phases of illness and therapeutic agents. Virol J.

[31] Ramasamy S, Subbian S. Critical determinants of cytokine storm and Type I interferon response in COVID-19 pathogenesis. 2021;34:e00299–20. 10.1128/CMR.00299-20 PMC8142516 33980688

[32] Karki R, Sharma BR, Tuladhar S, Williams EP, Zalduondo L, Samir P (2021). Synergism of TNF-α and IFN-γ Triggers Inflammatory Cell Death, Tissue Damage, and Mortality in SARS-CoV-2 Infection and Cytokine Shock Syndromes. Cell.

[33] Kumar D, Trivedi N (2021). Disease-drug and drug-drug interaction in COVID-19: Risk and assessment. Biomed Pharmacother.

[34] Ma Q, Li R, Pan W, Huang W, Liu B, Xie Y (2020). Phillyrin (KD-1) exerts anti-viral and anti-inflammatory activities against novel coronavirus (SARS-CoV-2) and human coronavirus 229E (HCoV-229E) by suppressing the nuclear factor kappa B (NF-κB) signaling pathway. Phytomedicine.

[35] Kandasamy M (2021). NF-κB signalling as a pharmacological target in COVID-19: potential roles for IKKβ inhibitors. Naunyn Schmiedebergs Arch Pharm.

[36] Adamsick ML, Gandhi RG, Bidell MR, Elshaboury RH, Bhattacharyya RP (2020). Remdesivir in patients with acute or chronic kidney disease and COVID-19. J Am Soc Nephrol.

[37] Mahase E (2021). Covid-19: Pfizer’s paxlovid is 89% effective in patients at risk of serious illness, company reports. BMJ.

[38] Ling J, Kumar R (2012). Crosstalk between NFkB and glucocorticoid signaling: a potential target of breast cancer therapy. Cancer lett.

[39] Didonato JA, Saatcioglu F, Karin M (1996). Molecular mechanisms of immunosuppression and anti-inflammatory activities by glucocorticoids. Am J Respir Crit Care Med.

[40] Atal S, Fatima Z (2020). IL-6 inhibitors in the treatment of serious COVID-19: a promising therapy?. Pharm Med.

[41] Marconi VC, Ramanan AV, de Bono S, Kartman CE, Krishnan V, Liao R (2021). Efficacy and safety of baricitinib for the treatment of hospitalised adults with COVID-19 (COV-BARRIER): a randomised, double-blind, parallel-group, placebo-controlled phase 3 trial. Lancet Respir Med.

[42] Kokkotis G, Kitsou K, Xynogalas I, Spoulou V, Magiorkinis G, Trontzas I (2022). Systematic review with meta-analysis: COVID-19 outcomes in patients receiving anti-TNF treatments. Aliment Pharm Ther.

[43] Kyriazopoulou E, Poulakou G, Milionis H, Metallidis S, Adamis G, Tsiakos K (2021). Early treatment of COVID-19 with anakinra guided by soluble urokinase plasminogen receptor plasma levels: a double-blind, randomized controlled phase 3 trial. Nat Med.

[44] Meng M, Zhao Q, Kumar R, Bai C, Deng Y, Wan B (2020). Impact of cardiovascular and metabolic diseases on the severity of COVID-19: a systematic review and meta-analysis. Aging.

[45] Zhao H, To KKW (2021). Cross-linking peptide and repurposed drugs inhibit both entry pathways of SARS-CoV-2. Nat Commun.

[46] Oroojalian F, Haghbin A, Baradaran B, Hemmat N, Shahbazi MA, Baghi HB (2020). Novel insights into the treatment of SARS-CoV-2 infection: An overview of current clinical trials. Int J Biol Macromol.

[47] Kamyshnyi O, Matskevych V, Lenchuk T, Strilbytska O, Storey K, Lushchak O (2021). Metformin to decrease COVID-19 severity and mortality: Molecular mechanisms and therapeutic potential. Biomed Pharmacother.

[48] Lenze EJ, Mattar C, Zorumski CF, Stevens A, Schweiger J, Nicol GE (2020). Fluvoxamine vs Placebo and Clinical Deterioration in Outpatients With Symptomatic COVID-19: A Randomized Clinical Trial. JAMA.

[49] Axfors C, Schmitt AM (2021). Mortality outcomes with hydroxychloroquine and chloroquine in COVID-19 from an international collaborative meta-analysis of randomized trials. Nat Commun.

[50] RECOVERY Collaborative Group. (2022). Aspirin in patients admitted to hospital with COVID-19 (RECOVERY): a randomised, controlled, open-label, platform trial. Lancet.

[51] de Alencar JCG, Moreira CL, Müller AD, Chaves CE, Fukuhara MA, da Silva EA (2021). Double-blind, Randomized, Placebo-controlled Trial With N-acetylcysteine for Treatment of Severe Acute Respiratory Syndrome Caused by Coronavirus Disease 2019 (COVID-19). Clin Infect Dis.

[52] Hamidi SH, Kadamboor Veethil S, Hamidi SH (2021). Role of pirfenidone in TGF-β pathways and other inflammatory pathways in acute respiratory syndrome coronavirus 2 (SARS-Cov-2) infection: a theoretical perspective. Pharm Rep.

[53] Schönrich G, Raftery MJ, Samstag Y (2020). Devilishly radical NETwork in COVID-19: Oxidative stress, neutrophil extracellular traps (NETs), and T cell suppression. Adv Biol Regul.

[54] Horby P, Lim WS, Emberson JR, Mafham M, Bell JL, Linsell L (2021). Dexamethasone in Hospitalized Patients with Covid-19. N Engl J Med.

[55] Usmani SZ, Quach H, Mateos MV, Landgren O, Leleu X, Siegel D (2022). Carfilzomib, dexamethasone, and daratumumab versus carfilzomib and dexamethasone for patients with relapsed or refractory multiple myeloma (CANDOR): updated outcomes from a randomised, multicentre, open-label, phase 3 study. Lancet Oncol.

[56] Elahi R, Karami P, Heidary AH, Esmaeilzadeh A (2022). An updated overview of recent advances, challenges, and clinical considerations of IL-6 signaling blockade in severe coronavirus disease 2019 (COVID-19). Int Immunopharmacol.

[57] Samuel SM, Varghese E, Büsselberg D (2021). Therapeutic Potential of Metformin in COVID-19: Reasoning for Its Protective Role. Trends Microbiol.

[58] Xian H, Liu Y, Rundberg Nilsson A, Gatchalian R, Crother TR, Tourtellotte WG (2021). Metformin inhibition of mitochondrial ATP and DNA synthesis abrogates NLRP3 inflammasome activation and pulmonary inflammation. Immunity.

[59] Scheen AJ (2020). Metformin and COVID-19: From cellular mechanisms to reduced mortality. Diabetes Metab.

[60] Zoulikha M, Huang F, Wu Z, He W (2022). COVID-19 inflammation and implications in drug delivery. J Control Release.

[61] https://www.who.int/health -topics/hypertension#tab=tab_1

[62] https://www.who.int/news -room/fact -sheets/detail/noncommunicable-diseases

[63] Davis HE, McCorkell L (2023). Long COVID: major findings, mechanisms and recommendations. Nat Rev Microbiol.

[64] Moukayed M (2023). A Narrative Review on the Potential Role of Vitamin D(3) in the Prevention, Protection, and Disease Mitigation of Acute and Long COVID-19. Curr Nutr Rep.

